# ROS-responsive nano-drug delivery system combining mitochondria-targeting ceria nanoparticles with atorvastatin for acute kidney injury

**DOI:** 10.7150/thno.40395

**Published:** 2020-01-16

**Authors:** Hui Yu, Feiyang Jin, Di Liu, Gaofeng Shu, Xiaojuan Wang, Jing Qi, Mingchen Sun, Ping Yang, Saiping Jiang, Xiaoying Ying, Yongzhong Du

**Affiliations:** 1 Institute of Pharmaceutics, College of Pharmaceutical Sciences, Zhejiang University, 866 Yu-Hang-Tang Road, Hangzhou, 310058, China.; 2 Department of Pharmacy, The First Affiliated Hospital, College of Medicine, Zhejiang University, 79 Qingchun Road, Hangzhou, 310003, China.

**Keywords:** acute kidney injury, oxidative stress, ROS-responsive, ceria, mitochondria-targeting.

## Abstract

Acute kidney injury (AKI) caused by sepsis is a serious disease which mitochondrial oxidative stress and inflammatory play a key role in its pathophysiology. Ceria nanoparticles hold strong and recyclable reactive oxygen species (ROS)-scavenging activity, have been applied to treat ROS-related diseases. However, ceria nanoparticles can't selectively target mitochondria and the ultra-small ceria nanoparticles are easily agglomerated. To overcome these shortcomings and improve therapeutic efficiency, we designed an ROS-responsive nano-drug delivery system combining mitochondria-targeting ceria nanoparticles with atorvastatin for acute kidney injury.

**Methods**: Ceria nanoparticles were modified with triphenylphosphine (TCeria NPs), followed by coating with ROS-responsive organic polymer (mPEG-TK-PLGA) and loaded atorvastatin (Atv/PTP-TCeria NPs). The physicochemical properties, *in vitro* drug release profiles, mitochondria-targeting ability, *in vitro* antioxidant, anti-apoptotic activity and *in vivo* treatment efficacy of Atv/PTP-TCeria NPs were examined.

**Results**: Atv/PTP-TCeria NPs could accumulate in kidneys and hold a great ability to ROS-responsively release drug and TCeria NPs could target mitochondria to eliminate excessive ROS. *In vitro* study suggested Atv/PTP-TCeria NPs exhibited superior antioxidant and anti-apoptotic activity. *In vivo* study showed that Atv/PTP-TCeria NPs effectively decreased oxidative stress and inflammatory, could protect the mitochondrial structure, reduced apoptosis of tubular cell and tubular necrosis in the sepsis-induced AKI mice model.

**Conclusions:** This ROS-responsive nano-drug delivery system combining mitochondria-targeting ceria nanoparticles with atorvastatin has favorable potentials in the sepsis-induced AKI therapy.

## Introduction

Acute kidney injury (AKI) is a heterogeneous clinical syndrome with variable pathogenesis and diverse outcomes 1. In critically ill patients, sepsis is the most common trigger of AKI, and AKI is a common complication of sepsis 2, with the incidence varies from 11% to 42% and may even reach 67% in the septic surgical population 3, which dramatically increases the mortality in patients with sepsis 4. Therefore, the alleviation of AKI could significantly improve the survival rate of patients with sepsis. Currently, there are no specific therapies to treat sepsis-induced AKI and clinicians could only rely on generalized supportive care 5.

Most research to date has shown that a common cause of sepsis-induced AKI is the development of the systemic inflammatory response syndrome mediated by increased oxidative stress 6. Other studies have pointed out that sepsis causes injury to kidney tubular cells by damaging the endothelium which leads to increased permeability 7. At the cellular level, polymicrobial insult may cause tubular cell death by caspase-mediated apoptosis 8. Various functional nanomaterials have been used to treat AKI, such as POM nanoclusters with the capability to scavenge detrimental ROS 9 and DNA origami, which have preferential renal uptake ability and efficacy similar to the antioxidant 10. It should be noted that mitochondria are the major intracellular source of ROS generation, the excessive production of ROS associated with mitochondrial dysfunction can lead to an imbalance between ROS production and the cells defense mechanism, which induce oxidative stress 11. Recent studies increasingly consider mitochondria to be involved in the pathophysiology of sepsis-induced AKI and can be used as a potential therapeutic target 12. Besides, due to the variable pathogenesis, multi-target therapy, not only the antioxidant may be more suitable for AKI.

It is well known that ceria nanoparticles (Ceria NPs) (<5 nm) exhibit the activity of ROS elimination in a recyclable way by reversible binding of oxygen atoms and shuttling between the Ce ^3+^and Ce ^4+^ states on the surface 13,14. The Ce ^3+^ sites are known to remove ROS *via* superoxide dismutase (SOD)-mimetics and remove**^.^**OH *via* redox reactions, while the Ce ^4+^ sites are responsible for the oxidation of H_2_O_2_ *via* catalase (CAT)-mimetics 15,16. Ceria NPs have been applied to treat ROS-related diseases such as Alzheimer's disease, sepsis and ischemic stroke 13,17,18. Nevertheless, there are remaining several shortcomings: (i) Ceria NPs with a diameter less than 5 nm are more favorable to ensure the high biomimetic enzyme activity 12,19, however, the ultra-small sizes will lead to a short half-life in the blood circulation, and the easy interparticle agglomeration of these ultra-small Ceria NPs will aggravate the problem 20,21. (ii) Mitochondria are critical intracellular loci of ROS production 22, but ordinary Ceria NPs can't selectively target mitochondria. (iii) Safety concerns related to the potential toxicity of Ceria NPs remain 23,24, the dosage of Ceria NPs should be minimized due to the potential toxicity.

Here, we report the design and synthesis of triphenylphosphine (TPP)-modified Ceria NPs with coating by mPEG-TK-PLGA polymer and simultaneously encapsulate atorvastatin (Atv/PTP-TCeria NPs). The enhanced permeability of vessels due to the inflammation in the kidney after AKI and suitable particle size could endow Atv/PTP-TCeria NPs with the ability to passively target the kidney 7,25,26. mPEG-TK-PLGA coating significantly improves the biocompatibility, mono-dispersity and prolongs the half-life in the bloodstream 20,27, moreover, allows NPs to load and responsively release drugs (the thioketal bond can be readily cleaved by ROS 28). Loading atorvastatin, which has been reported to have an ameliorative effect on acute kidney injury 29, 30, could help reduce the dosage of Ceria NPs and increase therapeutic effect and TCeria NPs would target mitochondria through TPP-derived activity to effectively eliminate excessive ROS.

## Materials and methods

### Materials

All chemical reagents were obtained from Sigma-Aldrich (St. Louis, MO) except as otherwise stated. mPEG (MW = 2.0 kDa), PLGA-COOR (70:30, MW = 1.5 kDa) were obtained from Dai Gang Biotechnology (Jinan, China), Anti-Caspase-3 antibodies were obtained from Abcam (UK). The ELISA kits of IL-6 and TNF-α were purchased from Boster Biotechnology (Wuhan, China). ICR male mice aged 8-10 weeks had 20-25 g weights were obtained from Zhejiang Medical Animal Centre (Hangzhou, China).

### Synthesis of Ceria and TCeria NPs

The previously reported procedures were used for the synthesis of TCeria nanoparticles with hydrolytic sol-gel reactions 11. Firstly, cerium (III) acetate (0.430 g, 1 mmol), triphenylphosphine (1.05 g, 4 mmol) and oleylamine (2.14 g, 8 mmol) were dissolved in 15 mL xylene, and the mixture solution was ultrasonic for 15 min. The next steps are the same as previously reported.

### Synthesis of TK and mPEG-TK-PLGA

TK was synthesized by typical synthesis method 31, to synthesize mPEG-TK-PLGA, TK (77.9 mg, 0.309 mmol), N, N'-dicyclohexyl carbodiimide (DCC) (381 mg,1.85 mmol), 4-dimethylaminopyridine (DMAP) (22.6 mg,0.185 mmol) (the molar ratio of the three feeding is 1:6:0.6), were dissolve in 15 ml anhydrous dimethyl sulfoxide, and stirred at 60 °C for 30 min to activate the carboxyl group of TK. Then 463 mg ester-terminated polylactic acid-glycolic acid copolymer (PLGA, 1.5 kDa, 70:30) dissolved in 4 mL anhydrous DMSO was added to the solution. The reaction continued at 60 °C under nitrogen protection. After 24 h, 618 mg methoxypolyethylene glycols (mPEG, 2 kDa) was dissolved in 4 mL anhydrous DMSO and added to the reaction system. The reaction continued at 60 °C for another 24 h under nitrogen atmosphere.

After that, the reaction mixture was extensively dialyzed (MWCO 7 kDa, Spectrum Laboratories, Laguna Hills, CA) against deionized water to remove DCC and DMAP. The polymer was obtained as white powders after freeze-drying under vacuum.

### Preparation of PTP-TCeria and Atv/PTP-TCeria NPs

mPEG-TK-PLGA polymer (10.0 mg) was dissolved in 10 mL of distilled water, then TCeria NPs (1.00 mg) was dissolved in 1 mL of DCM and added dropwise to polymer solution under vigorous stirring. Then the solution was ultrasound for 5 min to form an emulsion and stirred for 3 h to volatilize organic solvent, then centrifugated at 4000 rpm for 20 min to remove the agglomerates and bigger size nanoparticles.

To prepare the atorvastatin loaded PTP-TCeria NPs, atorvastatin was dissolved in 1 mL of the solution of acetone and methanol (1:1). Under vigorous stirring, the solution was added dropwise to the previously prepared PTP-TCeria NPs solution. Then the solution was stirred for 3 h to volatilize organic solvent, then centrifugated at 4000 rpm for 20 min to remove the free drug.

### Characterizations

* Measurement of hydrodynamic diameters and ζ-potentials*: Hydrodynamic diameters and ζ-potentials were measured by dynamic light scattering (DLS) (3000HS, Malvern Instruments Ltd.).

* Nuclear Magnetic Resonance (NMR)*: The structures of TK and mPEG-TK-PLGA were confirmed using a ^1^H NMR spectrometer (AC-80, Bruker Bios pin. Germany).

* Inductively coupled plasma mass spectrometry (ICP-MS) analysis*: [Ce] concentrations of samples were analyzed using inductively coupled plasma optical emission spectrometry (Agilent 725, Agilent Technologies, USA).

### *In vitro* drug release studies

* In vitro* atorvastatin release was studied using the dialysis method (PBS buffer, pH 7.4, containing 0/100/200 μM H_2_O_2_ as the dissolution medium). Put 1 mL Atv/PTP-TCeria NPs solution in a dialysis bag (MWCO3.5 kDa, CA) and dialyzed against 10 mL of dissolution medium under horizontal shaking (60 rpm) at 37 °C. After 0.5,1,2,4,6,8,12,24,36 h, collected the whole medium and replaced with fresh medium. Atorvastatin concentrations were determined using HPLC. The experiment was performed parallel in triplicate.

### Cytotoxicity studies

Human Umbilical Vein Endothelial Cells (HUVECs) were seeded at 96-well plates (2500 cells/well) and cultured for 24 h. TCeria NPs were diluted in cell media (increasing [Ce] concentrations: 0, 0.125, 0.250, 0.500, and 1.00 mM) and incubated with HUVECs. After incubating for 24 h, the viability was measured by MTT assay.

The cytotoxicity of mPEG-TK-PLGA polymer and PTP-TCeria NPs was determined by the same method. mPEG-TK-PLGA polymer were diluted in cell media (increasing concentrations :100, 200, 250, 300, 350, 400, 450 and 500 mg/L), PTP-TCeria NPs were diluted in cell media (increasing [Ce] concentrations: 0, 0.125, 0.250, 0.500, and 1.00 mM).

### Cellular uptake and mitochondria-targeting ability

HUVECs were seeded at 12-well plates (1x10^5^ cells/well) and cultured for 24 h. after 200 μM H_2_O_2_ stimulated for 2 h, the cells were then cultured with FITC-conjugated Ceria or TCeria NPs at 37 °C for different times (20,40,60,120 min). Then the cell was cultured with Mito-Tracker Orange CMTMRos for 30 min. TCeria NPs cellular uptake and mitochondria-targeting ability were observed using an LSM-780 microscope (Carl Zeiss, Oberkochen, Germany) and use Image J software to analyze the colocalization coefficients.

### *In vitro* antioxidant activity

* The protective effect of Atv/PTP-TCeria NPs from H_2_O_2_*: HUVECs were pretreated with H_2_O_2_ (200 μM) for 2 h and incubated with different interventions (atorvastatin, PTP-TCeria NPs, PTP and Atv/PTP-TCeria NPs) for 24 h. The cell viability was determined by MTT assays.

* Intracellular ROS measurement:* HUVECs were pretreated with H_2_O_2_ (200 μM) for 2 h and incubated with different interventions for 24 h, then incubated with DCFH-DA dye for 30 min. At last, cells were observed by fluorescence microscopy and took semi-quantitative analysis.

* SOD, MDA detection*: HUVECs were pretreated with H_2_O_2_ (200 μM) for 2 h and incubated with different interventions for 24 h. Then cells were lysed and centrifuged at 13000 rpm for 10 min and the supernatants were used as samples to measure the levels of SOD and MDA.

### *In vitro* anti-apoptotic activity

* Cell apoptosis*: HUVECs were pretreated with H_2_O_2_ (200 μM) for 2 h and incubated with different interventions for 24 h. The apoptosis effect of Atv/PTP-TCeria NPs was measured via Annexin V-FITC/PI apoptosis detection kit (Beyotime Biotech, China). At last, the cells were measured via flow cytometer.

* Mitochondrial Membrane Potential (Δψm) Measurement*: HUVECs were pretreated with H_2_O_2_ (200 μM) for 2 h and incubated with different interventions for 24 h. The mitochondrial membrane potential was measured using JC-1 kit assay (Beyotime Biotech, China). At last, cells were observed by fluorescence microscopy and took semi-quantitative analysis.

### AKI induction and treatment

All animal studies were conducted under IACUC approved protocols and in accordance with the Guide for the Care and Use of Laboratory Animals. We adopted an LPS-induced AKI mouse model 32, to study the therapeutic effect of Atv/PTP-TCeria NPs* in vivo*. The mice were intraperitoneally injected with LPS (2.00 mg/mL, 10.0 mg/kg) or saline and were divided randomly into the following groups: (1) control + saline group; (2) AKI +saline group; (3) AKI + atorvastatin; (4) AKI + PTP-TCeria NPs; (5)AKI + PTP; (6) AKI + Atv/PTP-TCeria NPs (for each group, n = 6). After that, animals received treatment by intravenous injection from the caudal vein (the dose of Ceria NPs was determined as 1.00 mg/kg). After 24 or 48 h, collected blood samples and kidneys for the following measurements.

* In vivo distribution*: AKI or healthy mice were injected intravenously with indocyanine green (ICG)-tetrabutylammonium iodide complex loaded PTP-TCeria NPs at a dose of 1.00 mg/kg. The mice were sacrificed at a predetermined time (3, 6, 9, 12, 24 and 36 h) and major organs were collected. Fluorescence signals in the collected organs were analyzed using the Maestro *in vivo* imaging system (Cambridge Research &Instrumentation, Inc., Woburn, MA, USA).

* Histological analysis:* Kidney tissues were fixed in 4.5% formalin buffer and embedded in paraffin. 5 micrometer thick sections were stained with hematoxylin and eosin (H&E), then examined using optical microscopy.

* Mitochondrial structure observation*: Kidney tissue was fixed in glutaraldehyde (2.5%), following fixed in osmium tetroxide (1%). Then the samples were dehydrated in graded alcohols and embedded in epoxy resin. After staining, sections were observed by transmission electron microscope.

## Results and discussion

### Synthesis and characterization of TCeria NPs

**Figure 1:**
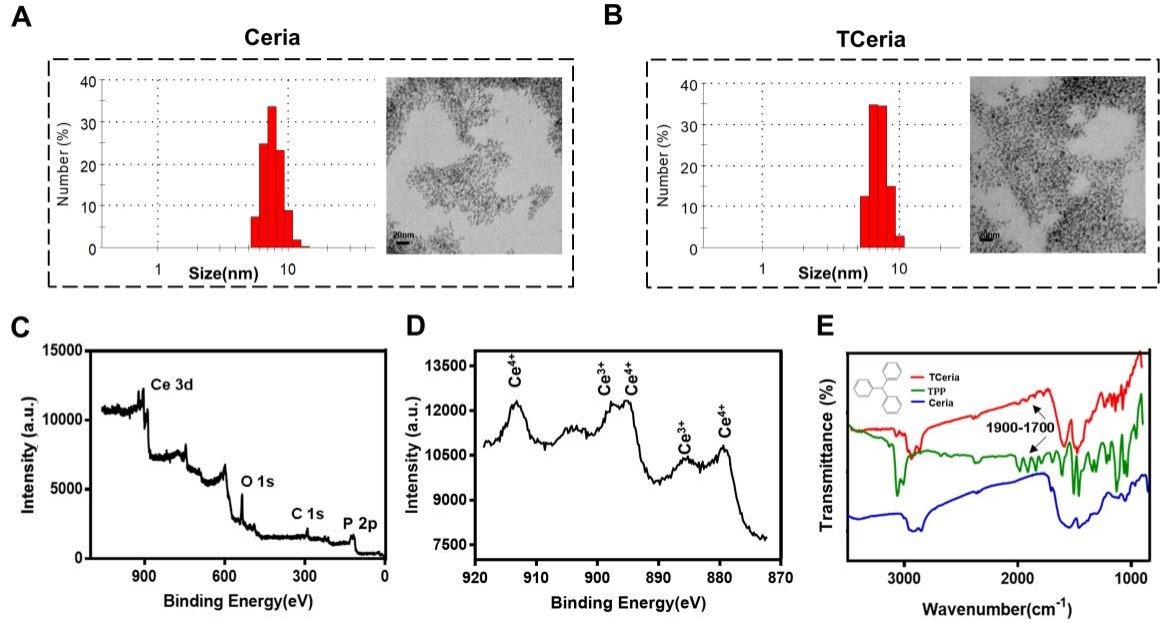
Synthesis and characterization of TCeria NPs. (A) Size distributions and TEM images (scale bar = 20 nm) of Ceria NPs and (B) TCeria NPs. (C) XPS spectrum of TCeria NPs. (D) XPS analysis of Ce 3d, Peaks at 881 and 887 eV are related to Ce ^4+^ and Ce ^3+^, peak at 915 eV indicates the presence of Ce ^4+^. (E) FT-IR spectrums of TPP, Ceria NPs, and TCeria NPs.

The uniform-sized ceria nanoparticles (Ceria NPs) and triphenylphosphine-modified ceria nanoparticles (TCeria NPs) were synthesized using a Sol-Gel Synthesis method 13. The hydrodynamic diameters of synthesized Ceria NPs and TCeria NPs were quite similar, with the average sizes of 7.74±1.35 nm, and 8.16±1.98 nm, respectively. Transmission electron microscopy (TEM) showed that both of Ceria NPs and TCeria NPs had uniform morphologies (Figure 1A and B). As shown by the XPS analysis results, Figure 1C exhibited both specific peaks of Ce and Phosphorus (around 900 and 130 eV), which confirmed the successful modification of triphenylphosphine on the surface of Ceria NPs. Figure 1D showed the peaks of both Ce ^3+^ and Ce ^4+^ oxidation states, which are necessary for the redox activity of Ceria NPs. The FI-TR examination displayed that TCeria NPs had the spectrum peaks of 1700~1900 cm^-1^, which were not found in the spectrum of Ceria NPs, this result suggested the presence of benzene ring in TCeria NPs, also indicating the successful modification of triphenylphosphine on Ceria NPs (Figure 1E). Such modification is important for Ceria NPs to target mitochondria to eliminate the excessive ROS since the strategy of using TPP to improve the antioxidant defenses of mitochondria has been reported to be feasible and promising 33,34.

### Synthesis and characterization of Atv/PTP-TCeria NPs

**Figure 2:**
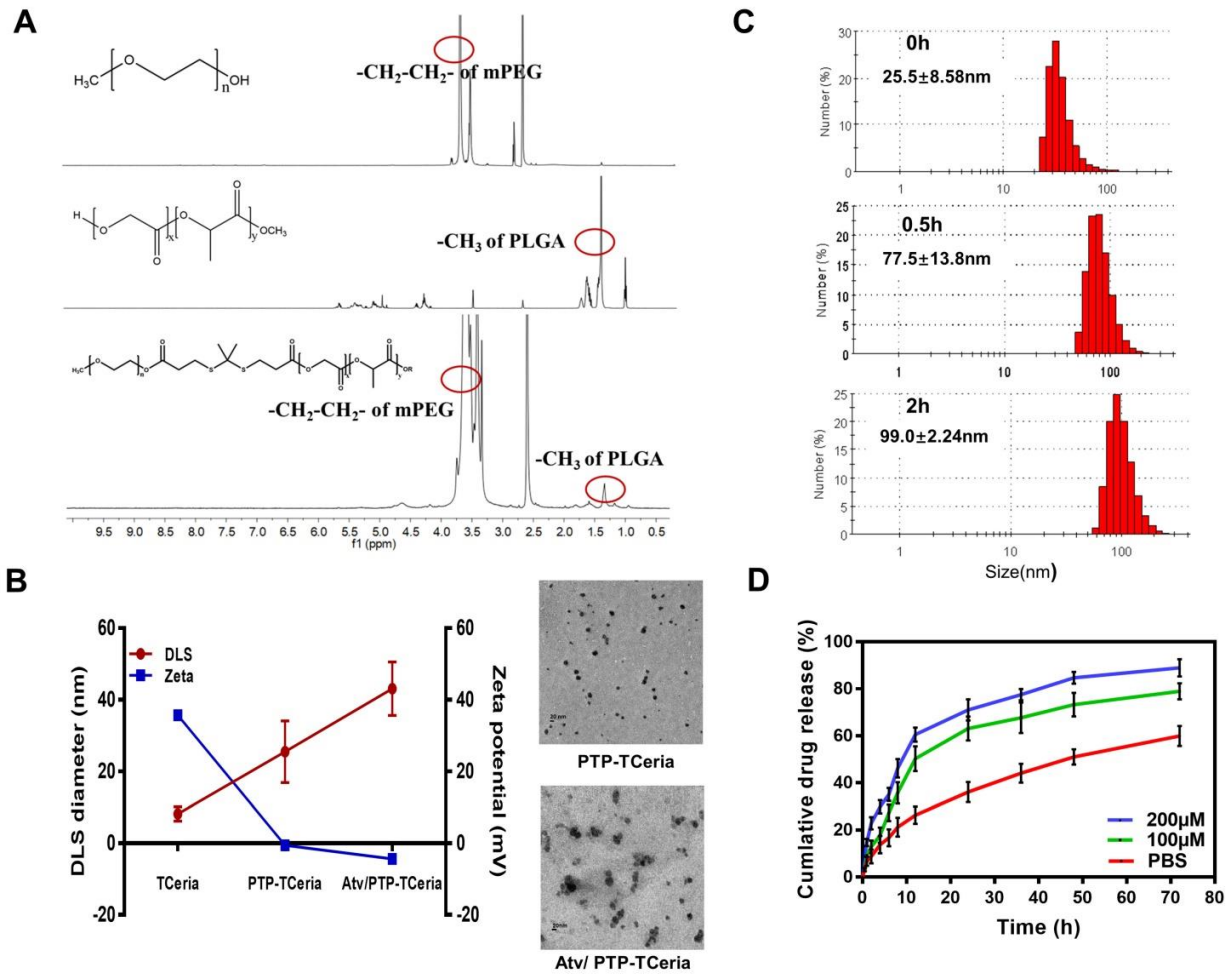
Synthesis and characterization of Atv/PTP-TCeria NPs. (A) ^1^H-NMR spectra of mPEG, PLGA, and mPEG-TK-PLGA from top to bottom, respectively. (B) Hydrodynamic diameter distributions and zeta potentials of TCeria, PTP-TCeria, Atv/PTP-TCeria NPs and TEM images of PTP-TCeria and Atv/PTP-TCeria NPs dispersed in DI water, scale bar = 20 nm. (C) Diameter changes of PTP-TCeria NPs cultivated with 100 mM H_2_O_2_. (D) Drug release behaviors of Atv/PTP-TCeria NPs. The release of atorvastatin was observed with time in the presence of 0 μM/100 μM/ 200 μM H_2_O_2_ in PBS buffer at pH 7.4.

mPEG-TK-PLGA polymer was synthesized by esterification of the carboxyl group of TK 35 with the hydroxy group of mPEG and PLGA. After the successful formation of the mPEG-TK-PLGA, the typical peak of mPEG and PLGA would appear in the^ 1^H NMR spectra of mPEG-TK-PLGA. Figure 2A showed the ^1^H NMR spectra of mPEG, PLGA, and mPEG-TK-PLGA and as can be seen, the characteristic signals of -CH_2_-CH_2_- in the mPEG segment and -CH_3_ in the PLGA segment were located at ~3.5 and ~1.5 ppm, respectively. These results demonstrated the successful formation of mPEG-TK-PLGA. Besides, a detailed analysis was shown in Figure S2B.

The presence of the mPEG-TK-PLGA coating on TCeria NPs is very crucial to make the nanoparticles stable, biocompatible, efficiently encapsulate and responsively release therapeutic drugs 36. After coated with mPEG-TK-PLGA, the hydrodynamic diameter of TCeria NPs was increased from 8.16±1.98 nm to 25.5±8.58 nm and the ζ-potential was changed from positive (+35.7±0.630 mV) to negative (-0.551±0.114 mV). The changes in size and surface potential confirmed the successful coating of mPEG-TK-PLGA on the nanoparticles. After loaded the drug, the hydrodynamic diameter of PTP-TCeria NPs was increased to 43.1±7.50 nm and the ζ-potential was changed to -4.45±0.751 mV. Moreover, TEM images indicated both of PTP-TCeria NPs and Atv/ PTP-TCeria NPs had round and relatively homogeneous morphology (Figure 2B). It is reported that nanoparticles with sizes of 30 ~ 80 nm can target the mesangium of kidney 25,37. Therefore, Atv/ PTP-TCeria NPs have a suitable particle size and are expected to distribute more in the kidneys. Besides, there are lots of factors that would affect the stability of colloids, such as solvation and steric stabilization caused by PEG 38,39 and the zeta-potential is just one of them. Although the ζ-potential of Atv/PTP-TCeO2 NPs was quite small, the diameters of Atv/PTP-TCeria NPs were no significant difference after storing for 15 days in 4°C, showing it had good stability (Figure S3).

What's more, mPEG-TK-PLGA polymer is expected to have ROS-responsive ability due to the presence of the TK-containing linker 35,40,41. To evaluate the ROS-responsive ability of mPEG-TK-PLGA, the diameters of PTP-TCeria NPs cultivated with 100 mM H_2_O_2_ were measured in a predetermined time. The result showed that the diameters of PTP-TCeria NPs were changed from 25.5±8.58 nm to 99.0±2.24 nm after cultivated with H_2_O_2_ for 2 h, indicating that the mPEG-TK-PLGA had great ROS-responsive ability due to the thioketal bond which can be readily cleaved by ROS (Figure 2C).

Besides, *in vitro* release studies of atorvastatin were investigated in different conditions. In the high H_2_O_2_ concentration condition (pH 7.40 PBS buffer containing 200 μM H_2_O_2_), Atv/PTP-TCeria NPs released drug rapidly in the first 12 h, in which 60.5±2.89% of drug was released (Figure 2D). Notably, in the normal condition (pH 7.40 PBS buffer containing 0 μM H2O2), Atv/PTP-TCeria NPs presented much slower drug release, with only 26.3±3.67% of drug was released in the first 12 h. These results demonstrated that Atv/PTP-TCeria NPs had a good ability to ROS-responsive release drug.

### Cellular uptake and mitochondria-targeting ability of TCeria NPs

**Figure 3:**
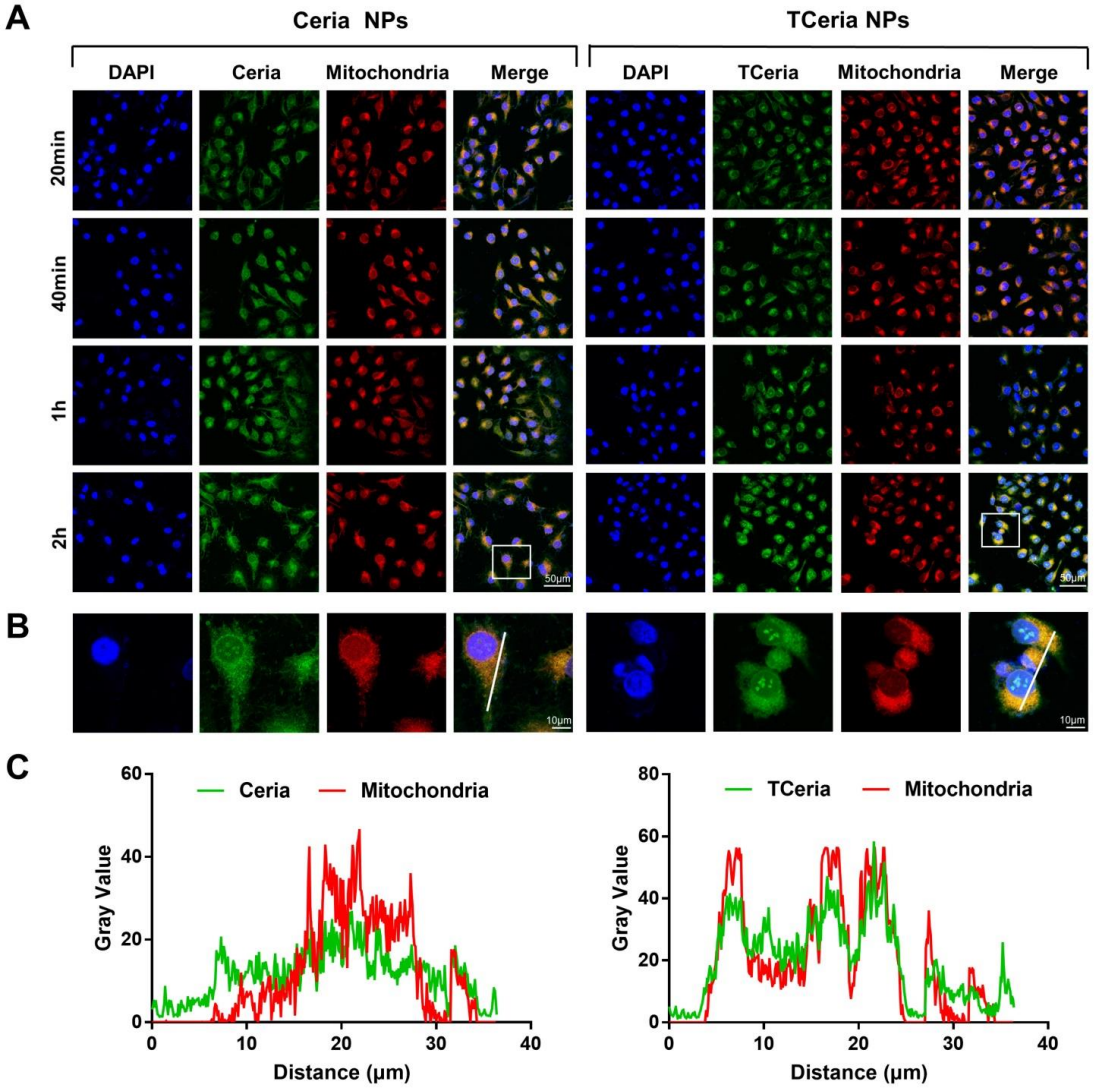
Cellular uptake and mitochondria-targeting ability of TCeria NPs. (A) Images of HUVECs treated with FITC-Ceria NPs, FITC-TCeria NPs at a dose of 0.100 mM for 20 min, 40 min,1 h and 2 h with mitochondria stained, scale bar = 50 μm. (B) Images of HUVECs treated with FITC-Ceria NPs, FITC-TCeria NPs for 2 h with mitochondria stained, scale bar = 10 μm. (C) The colocalization analysis of the fluorescent intensity of Ceria NPs, TCeria NPs and mitochondria in cells from figure 3B.

H_2_O_2_-stimulated HUVECs were incubated with 0.100 mM of FITC-TCeria NPs and FITC-Ceria NPs for the predetermined time points (20 min, 40 min, 1 h, and 2 h). As shown in Figure 3A**,** the fluorescence intensity of cells treated with FITC-TCeria NPs (green color) was high even after a short time of 20 min, indicating that the cellular uptake rate of TCeria NPs was fast. In addition to endocytosis, which usually takes longer, other mechanisms may be involved in the cellular uptake of the FITC-TCeria NPs. It has been reported that Ceria NPs enter the cytoplasm of epithelial cells in a size-dependent manner, the small size of nanoceria particles (3-5 nm) may allow other routes of entry, except for phagocytosis 42. We also observed that Ceria NPs had mitochondrial targeting ability to some extent since the fluorescence of Ceria NPs and mitochondria had some overlap (Figures 3B-C), the possible reason is that Ceria NPs had features for mitochondria targeting, such as small size and hydrophobicity 13. On the other hand, TCeria NPs presented a significantly higher colocalization coefficient in mitochondria than that of Ceria NPs, indicating that the TCeria NPs could more effectively target to mitochondria through TPP-derived targeting activity.

### *In vitro* activity of Atv/PTP-TCeria NPs

**Figure 4:**
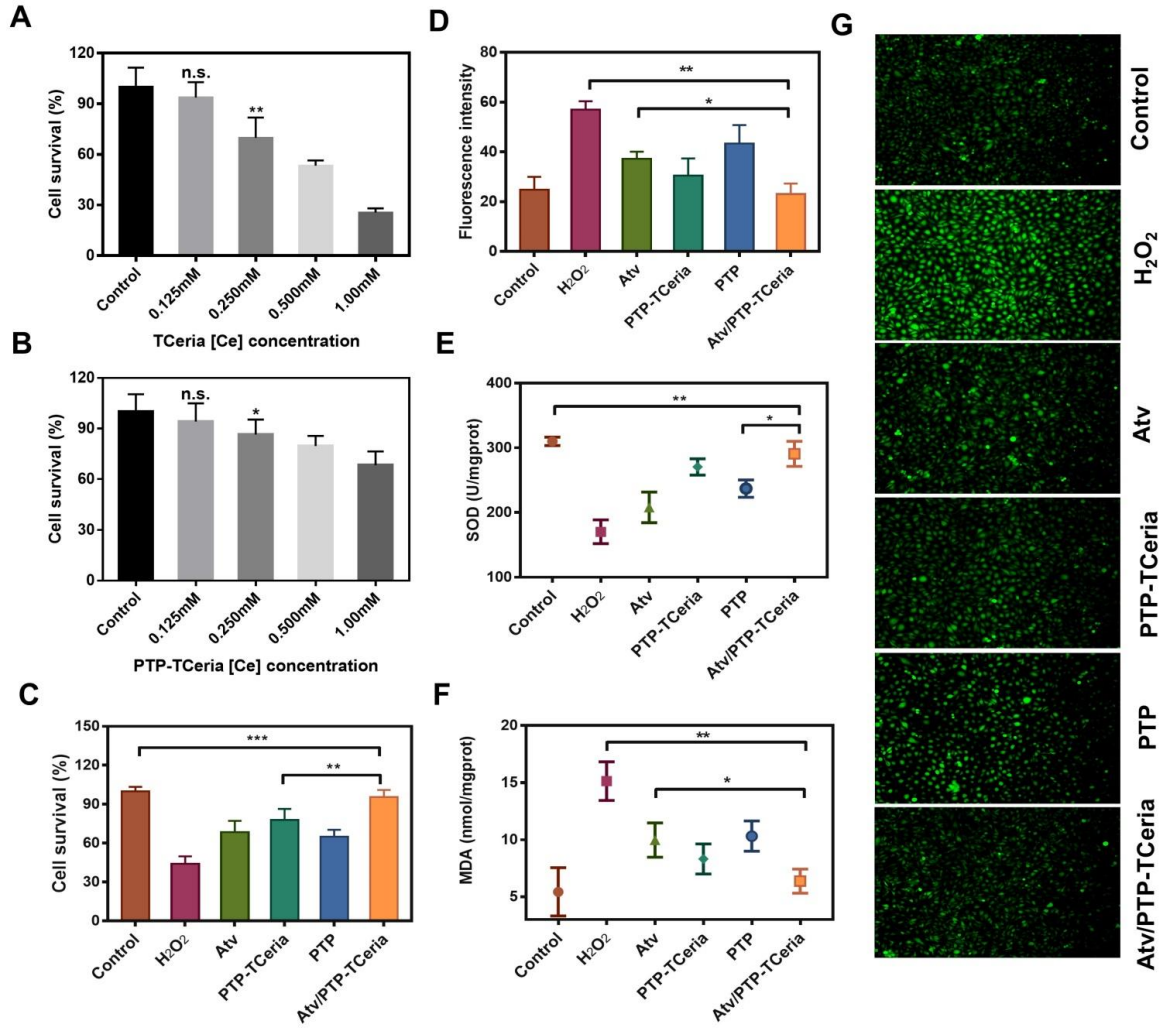
*In vitro* antioxidant capacity of Atv/PTP-TCeria NPs. (A) Cell viability of TCeria NPs, (B) PTP-TCeria NPs in HUVECs after 24 h exposure. (C) Cell viability of H_2_O_2_-stimulated HUVECs after incubated with atorvastatin, PTP-TCeria, PTP or Atv/PTP-TCeria NPs for 24 h. (D) Semi-quantitative results of intracellular ROS in figure 4g. (E) SOD activity and (F) MDA level of H_2_O_2_-stimulated HUVECs after incubated with atorvastatin, PTP-TCeria, PTP or Atv/PTP-TCeria NPs for 24 h. (G) Intracellular ROS of H_2_O_2_-stimulated HUVECs after incubated with atorvastatin, PTP-TCeria, PTP or Atv/PTP-TCeria NPs for 24 h was observed via fluorescent microscopy, scale bar = 100 μm. Data are expressed as the mean ± SD, n = 5 for each group.

The cytotoxicity of TCeria NPs, mPEG-TK-PLGA polymer, and PTP-TCeria NPs were assessed on HUVECs via MTT assay. As shown in Figure 4A and S4, TCeria NPs were nontoxic at [Ce] ion concentrations below 0.125 mM, and mPEG-TK-PLGA was nontoxic below 500 mg/L. At the same [Ce] ion concentrations, the viability of HUVECs cultivated with PTP-TCeria NPs was higher than those cultivated with TCeria NPs (Figure 4B), indicating that the toxicity of TCeria NPs could be reduced by coating with mPEG-TK-PLGA.

HUVECs stimulated by H_2_O_2_ were adopted as oxidative stress injured cell model to study antioxidative activity *in vitro* 43. Firstly, the effect of Atv/PTP-TCeria NPs on cell viability stimulated by H_2_O_2_ was investigated. As shown in Figure 4C, more than 50% of cells died after incubation with H_2_O_2_ for 24 h, The viability of H_2_O_2_-stimulated cells could be largely improved by incubating them with free atorvastatin, PTP-TCeria NPs, PTP and Atv/PTP-TCeria NPs. Notably, Atv/PTP-TCeria NPs increased cell survival most significantly and showed the best antioxidant effect compared to the other three interventions. (**p < 0.01).

Intracellular reactive oxygen species was measured using DCFH-DA probe. As shown in Figure 4D and G, the stronger green fluorescence intensity represented the higher concentration of ROS and images displayed that the fluorescent intensity in H_2_O_2_-stimulated cells treated with free atorvastatin/PTP-TCeria NPs / PTP or Atv/PTP-TCeria NPs was weaker than H_2_O_2_-stimulated cells without any intervention. The semi-quantitative results indicated that the green fluorescence intensity of H_2_O_2_-stimulated cells decreased to the lowest after incubated with Atv/PTP-TCeria NPs, which demonstrated that Atv/PTP-TCeria NPs had the better excessive ROS-scavenging ability than free atorvastatin and PTP(*p<0.05).

The* in vitro* antioxidant capacity of Atv/PTP-TCeria NPs was further investigated by assays of superoxide dismutase (SOD) and malondialdehyde (MDA). Compared with the H_2_O_2_-stimulated cells, Atv/PTP-TCeria NPs could significantly up-regulate SOD level (**p < 0.01) (Figure 4E) and down-regulate MDA level (**p < 0.01) in cells (Figure 4F) and the extent of regulation was more significant than free atorvastatin and PTP, which also demonstrated Atv/PTP-TCeria NPs had excellent antioxidant capacity (*p < 0.05).

According to the results of cell survival, ROS, SOD and MDA levels, Atv/PTP-TCeria NPs have the superior antioxidant capacity *in vitro*. The reason why Atv/PTP-TCeria NPs and PTP-TCeria NPs had no significant difference in assays results of ROS, SOD and MDA levels may be that the antioxidant effect of atorvastatin is much weaker when compared with TCeria NPs. Therefore, the antioxidant capacity of Atv/PTP-TCeria NPs was not significantly improved after loading atorvastatin.

**Figure 5:**
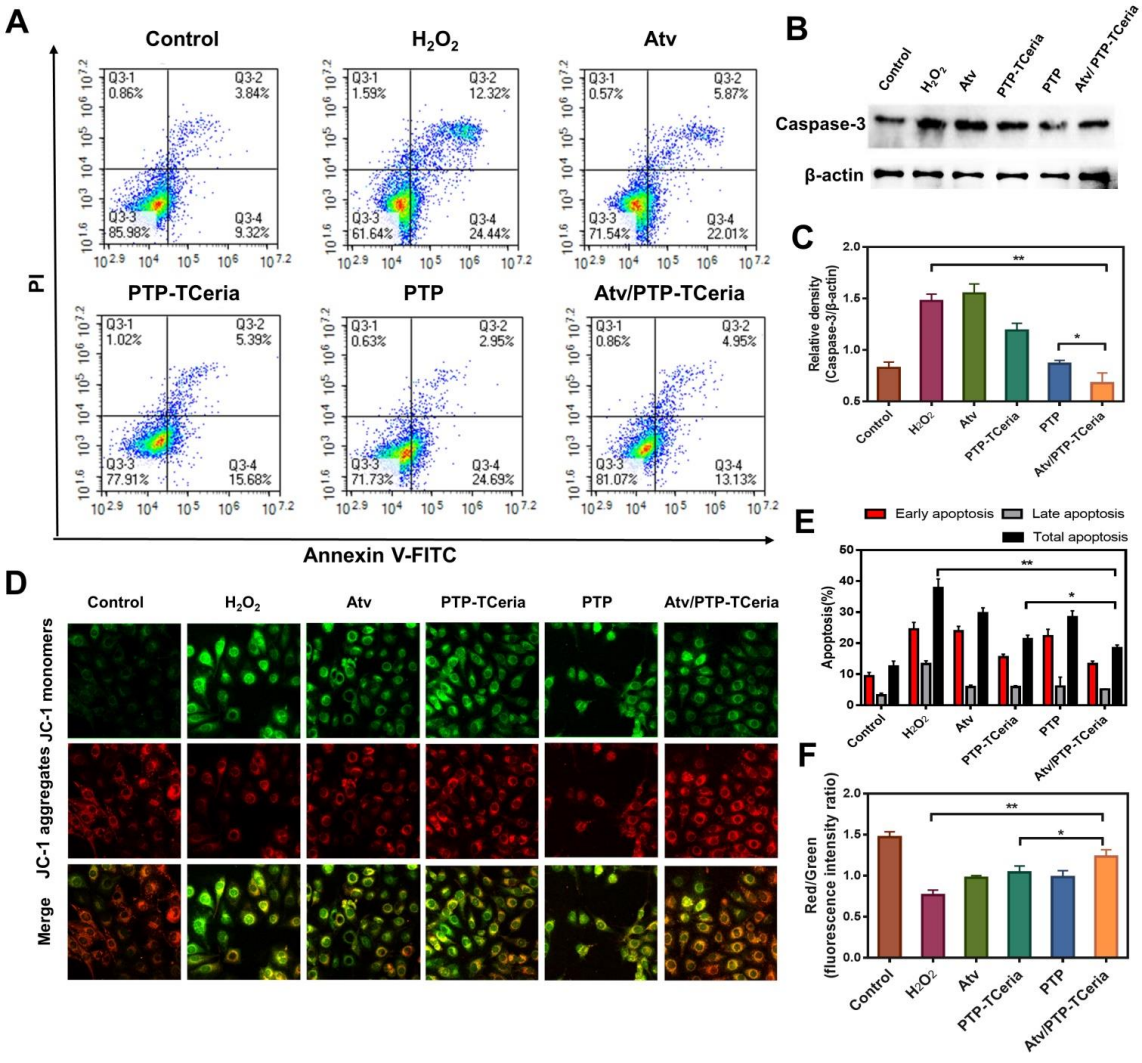
*In vitro* anti-apoptotic activity of Atv/PTP-TCeria NPs. H_2_O_2_-stimulated HUVECs were incubated with atorvastatin, PTP-TCeria, PTP and Atv/PTP-TCeria NPs for 24 h. (A) The Apoptosis in different groups was evaluated by flow cytometry. (B) The level of Caspase-3 in different groups was evaluated by western blot. (C) The semi-quantitative analysis of Caspase-3. (D) The Δψm of mitochondria in different groups was investigated using confocal microscopy, scale bar = 20 μm. (E) Semi-quantitative results of flow cytometry (F) Semi-quantitative results of the Δψm changes of mitochondria. Data are expressed as the mean ± SD, n = 3 for each group.

Additionally, apoptosis has been considered to play a role in the pathogenesis of sepsis-induced AKI 44, thus the *in vitro* anti-apoptotic effect of Atv/PTP-TCeria NPs was studied. Apoptotic cells were quantified using Annexin V-FITC/PI apoptosis detection kit. As Figure 5A showed, apoptotic cells were approximately 40% in the H_2_O_2_-stimulated group, but in Atv/PTP-TCeria NPs treated group, it decreased below 20%. The apoptotic cells reduction in Atv/PTP-TCeria NPs treated group was more significant than free atorvastatin, PTP-TCeria NPs and PTP treated groups (*p < 0.05) (Figure 5E), which indicated that Atv/PTP-TCeria NPs had a good anti-apoptosis effect on H_2_O_2_-stimulated cells model.

Caspase-3 is the most important terminal shear enzyme in the apoptotic process and was reported to be positively correlated with renal dysfunction 8. The expression level of Caspase-3 was determined by western blot analyses. The results showed that in H_2_O_2_-stimulated cells, the expression level of Caspase-3 was increased. Notably, treatment with Atv/PTP-TCeria NPs decreased the expression of Caspase-3, suggesting that Atv/PTP-TCeria NPs could inhibit apoptosis in H_2_O_2_-stimulated cells. (**p < 0.01) (Figure 5B-C). In addition, mPEG-TK-PLGA polymer also reduce the level of Caspase-3, probably because mPEG-TK-PLGA polymer contains the TK linker which could be oxidized by H_2_O_2_, the mPEG-TK-PLGA polymer have the ROS scavenging capacity and could relieve oxidative stress to some extent, which may affect the expression level of caspase-3 45,46.

Mitochondria are involved in apoptosis and a decrease in the membrane potential (Δψm) is considered to be an early marker of apoptosis 47, we used JC-1 staining method to measure the Δψm of HUVECs. The higher ratio of JC-1 red/green means the lower degree of mitochondria dysfunction. The Δψm of HUVECs was substantially decreased after H_2_O_2_ stimulated, however, the ratio of JC-1 red/green in Atv/PTP-TCeria NPs treated group was close to the control group and significantly higher than atorvastatin, PTP-TCeria NPs and PTP treated groups (Figure 5D and F, *p < 0.05), suggesting that Atv/PTP-TCeria NPs had the best ability to maintain normal membrane potential.

### * In vivo* efficacy of Atv/PTP-TCeria NPs

**Figure 6:**
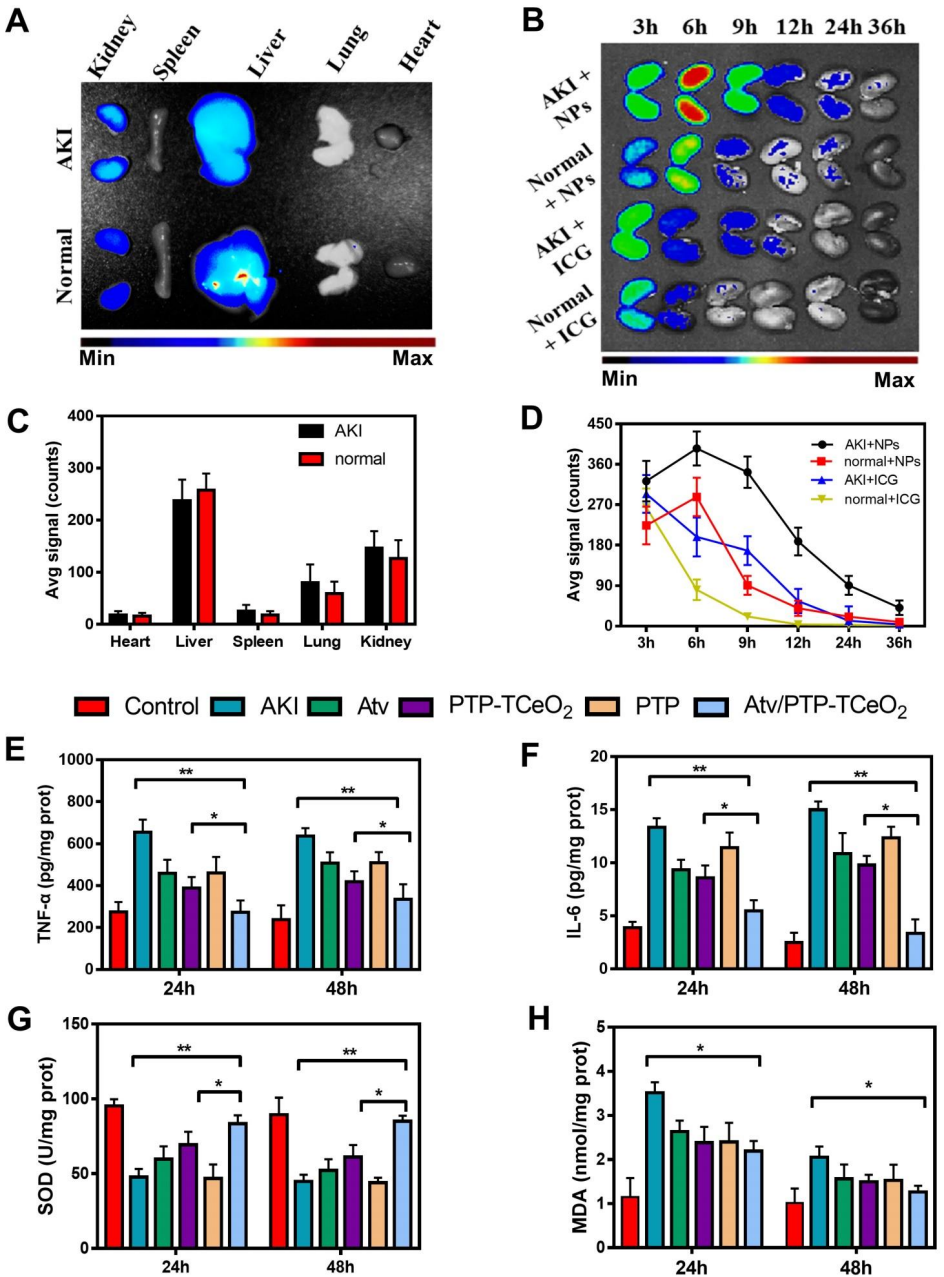
*In vivo* biodistribution and anti-inflammatory, antioxidant activity of Atv/PTP-TCeria NPs. (A) AKI mice were injected intravenously with ICG loading PTP-TCeria (1 mg/kg) and visualized at 6h after administration. (B) The kidney fluorescence intensity in ICG loading PTP-TCeria NPs and free ICG treated AKI mice model at different times. (C) The semi-quantitation of fluorescence intensity in figure 6a. (D) The semi-quantitation of fluorescence intensity in figure 6b. (E) The levels of TNF-α, (F) IL-6, (G) SOD, (H) MDA in the kidney at 24 h and 48 h after different treatments.

Firstly, the distribution of Atv/PTP-TCeria NPs in mice was examined. As shown in Figure 6A, liver and kidney had much higher fluorescence signal than other organs, which indicated Atv/PTP-TCeria NPs were mainly distributed in the liver and kidney due to passive targeting. Compared to healthy mice, AKI mice had higher kidney fluorescence signals because of the EPR effect in inflammation tissues.

In AKI or healthy mouse model, the kidney accumulation of ICG loaded PTP-TCeria NPs at different time points (3, 6, 12, 24, and 36 h) was shown in Figure 6B. The fluorescence signals of free ICG in kidneys were weak after 6h and the fluorescence signals of NPs in kidneys lasted for 36h, indicating that free ICG had much faster renal clearance rate than ICG loaded PTP-TCeria NPs. The results demonstrated that the overwhelming majority of the renal fluorescence signal after injection with NPs was because of NPs-encapsulated dye. It was observed that the fluorescence signals of AKI mice kidneys at each monitoring point were significantly brighter than those of healthy mice, which was because the renal clearance rate became lower due to kidney damage. As can be seen from the quantitative values of fluorescence intensity of different mice in Figure 6D, the accumulation of ICG loaded PTP-TCeria NPs in the kidneys of AKI model mice was higher than that of healthy mice, which was consistent with the previous report that the enhanced permeability of vessels due to the inflammation in the kidney could increase the accumulation of NPs in the kidneys 7.

Also, ICP was used to measure the amount of Ceria NPs in the main organs. As shown in Figure S7, the injected Ceria NPs were mainly distributed in the liver and kidneys (6.47±0.613 μg/g and 2.12±0.459 μg/g, respectively). It is reported the ultrasmall Ceria NPs with ~4.5 nm exhibited low renal accumulation but high liver accumulation because most of the injected nanomaterials are sequestered by the mononuclear phagocyte system (MPS) 48, however, mPEG-TK-PLGA polymer medication could effectively reduce the rapid uptake and clearance of nanoparticles by the MPS *in vivo* 49 and after the coated of mPEG-TK-PLGA polymer in our study, the dimeters of PTP-TCeria NPs was 43.1±7.50 nm which was suitable to distribute more in kidneys 25,37.

The anti-inflammatory efficacy of Atv/PTP-TCeria NPs was investigated and TNF-α, IL-6 levels were chosen as indexes for evaluating the severity of inflammatory. As can be seen from the Figure 6E-F, the group treated with Atv/PTP-TCeria NPs exhibited a significant decrease in TNF-α, IL-6 levels compared with the AKI group and Atv/PTP-TCeria NPs had better efficacy to reduce inflammatory factors than free atorvastatin, PTP-TCeria NPs, and PTP. (*p <0.05).

Next, the levels of SOD and MDA were measured to investigate the effects of Atv/PTP-TCeria NPs on oxidative stress. As shown in Figure 6G, the level of SOD significantly reduced in the AKI group. All treatments could increase the SOD level and Atv/PTP-TCeria NPs showed the best therapeutic efficacy compared with the other three groups (*p < 0.05).The level of MDA was increased in the AKI group, and was notably decreased in the group treated with Atv/PTP-TCeria NPs (*p < 0.05) (Figure 6H**)**. These two results mutually support the favorable antioxidant capacity of Atv/PTP-TCeria NPs.

**Figure 7:**
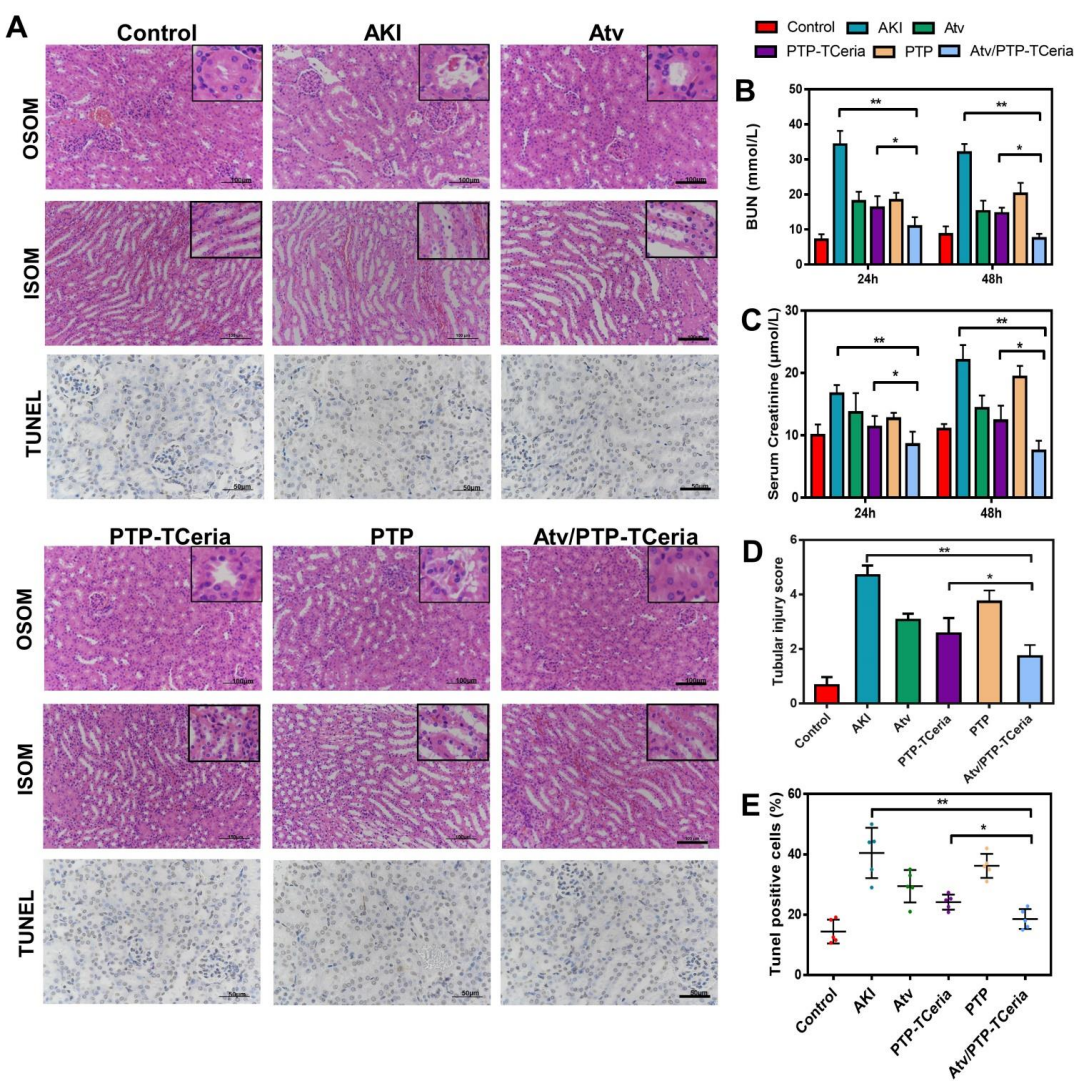
Histopathological changes, assessment on kidney function after Atv/PTP-TCeria NPs therapy and anti-apoptotic efficacy of Atv/PTP-TCeria NPs. (A) Hematoxylin and eosin (H&E)-stained kidney sections of the AKI mice after different treatments (atorvastatin, PTP-TCeria NPs, PTP and Atv/PTP-TCeria NPs) (at 48 h), Scale bar = 100 μm. Apoptosis cells staining using TUNEL assay (brown nuclei) of different groups (at 48 h), Scale bar = 50 μm. (b and c) Scrum and BUN levels at 24 and 48 h in the AKI mice after different treatments. (D) Cell necrosis scores. (E) The quantitation of TUNEL-positive cells.

Serum creatinine and blood urea nitrogen (BUN) are the most widely used parameter for evaluating the severity of renal dysfunction. The higher serum creatinine and BUN in serum level, the weaker kidney function 50,51. The levels of serum creatinine and BUN were significantly reduced after 24 h and 48 h in every treatment group compared with the AKI groups without any treatment (Figure 7B-C). Notably, serum creatinine and BUN levels in Atv/PTP-TCeria NPs treated group were the closest to the control group compared with the other three groups (*P <0.05), which suggested Atv/PTP-TCeria NPs had pleasurable effects to alleviate renal damage.

Hematoxylin and eosin (H&E) staining showed that kidney structure in the control group was normal, and brush borders visible in the proximal tubules in the outer stripe of outer medulla (OSOM) (Figure 7A). The kidneys from the AKI group had severe tubular damage in the OSOM, which were characterized by extensive luminal congestion and tubular necrosis. Compared with the AKI group, the renal damage of Atv/PTP-TCeria NPs treated groups was improved, where the renal tubular necrosis in the OSOM was significantly reduced, and the proximal tubule preserved the brush border, as well as tubular structure, was preserved in the inner stripe of outer medulla (ISOM). The results of cell necrosis scores are consistent with the observation, demonstrating the Atv/PTP-TCeria NPs had the best capacity to maintain the normal renal structure. (*p < 0.05) (Figure 7D).

*In vitro* experiments have shown that Atv/PTP-TCeria NPs held anti-apoptotic effect. Whether Atv/PTP-TCeria NPs could alleviate renal tubular apoptosis was also studied on the AKI mice model* via* TUNEL staining method. As can be seen in Figure 7A, the kidney of AKI mice had extensive nuclear changes consistent with apoptotic cell death, while there were few TUNEL-positive cells in the kidney of the control group. All treatments significantly reduced the percentage of TUNEL-positive cells, and Atv/PTP-TCeria NPs showed the best anti-apoptosis effect (*p < 0.05) (Figure 7E).

Mitochondria morphology change is an early sign of ROS-induced mitochondrial dysfunction 52. Transmission electron microscopy showed extensive mitochondrial damage in proximal tubular cells of representative samples obtained from the AKI group, which some mitochondria lost cristae and some mitochondria show rupture of the cell membrane and release of matrix material into the cytoplasm. In contrast, representative samples of Atv/PTP-TCeria NPs treated AKI mice showed many mitochondria, which retained cristae structure in intact invagination of basal membrane of tubular cells, suggesting that Atv/PTP-TCeria NPs had a good protective effect on mitochondrial structure.

**Figure 9:**
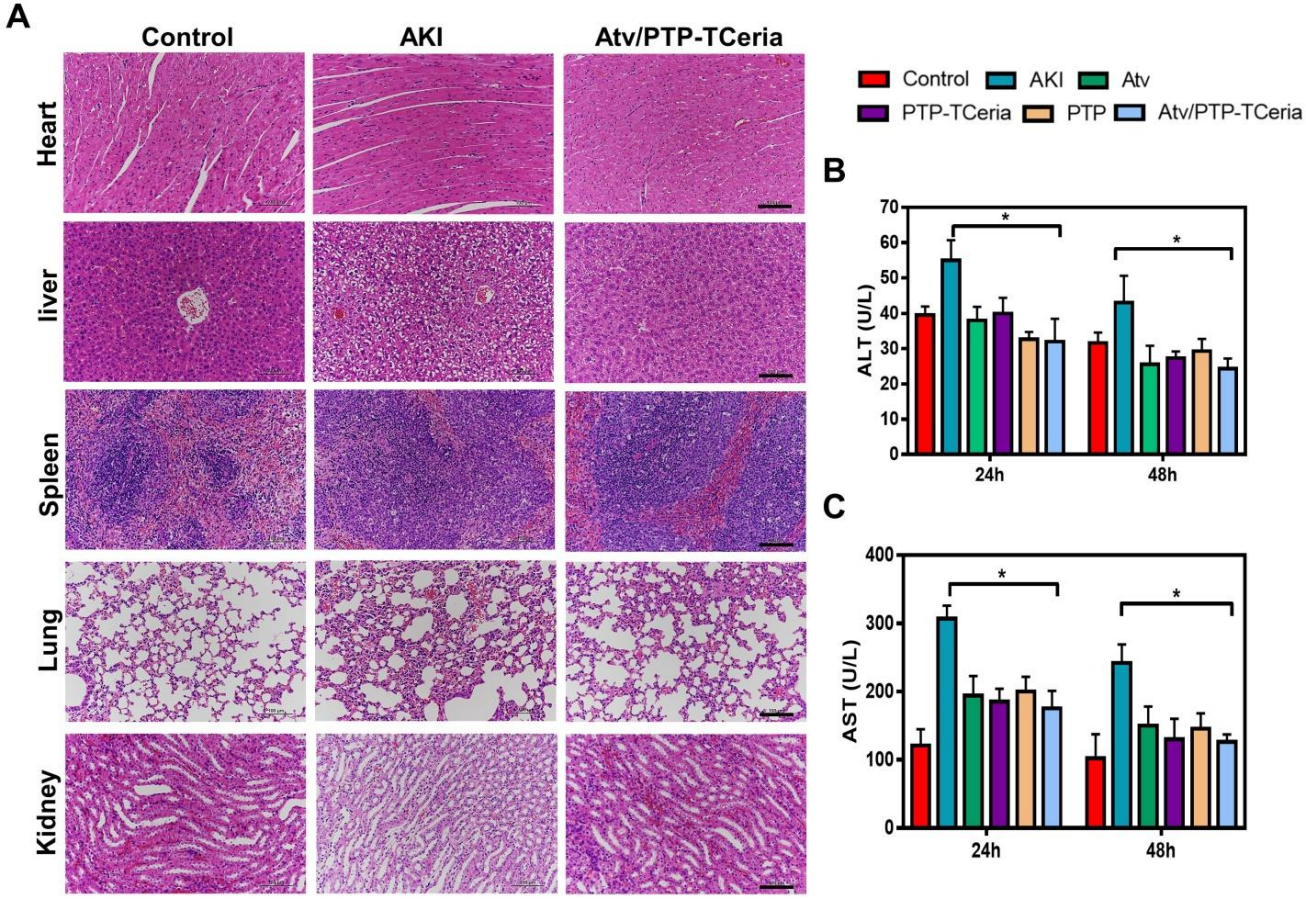
The evaluation of the adverse effects of different treatments. (A) Histopathology images of heart, liver, spleen, lung, and kidney collected from the control, AKI and Atv/PTP-TCeria NPs treated groups (at 48 h), scale bar = 100 μm. Concentrations of (B) ALT, (C) AST in the AKI mice after different treatments (atorvastatin, PTP-TCeria NPs, PTP and Atv/PTP-TCeria NPs). *p<0.05 compared with the AKI group.

Besides, it is necessary to evaluate the adverse effects of different treatments on the liver since the Atv/PTP-TCeria NPs were mainly distributed in the liver and kidney. As shown in Figure 9B-C, the levels of aspartate aminotransferase (AST) and alanine aminotransferase (ALT) in the AKI group were increased, but there was no significant difference between the control group and Atv/PTP-TCeria NPs treated group, indicating that LPS could cause liver damage in mice, and Atv/PTP-TCeria NPs had little adverse effect on the liver, even had therapeutic effect on liver damage caused by LPS. Moreover, H&E staining was performed to the major organs collected from each group. There was no significant damage in other major organs collected from the Atv/PTP-TCeria NPs groups which indicated that Atv/PTP-TCeria NPs had very few adverse effects on the major organs and may even help relieve liver and lung damage caused by LPS (Figure 9A).

## Conclusion

In summary, the ROS-responsive nano-drug delivery system (Atv/PTP-TCeria NPs), composed of monodispersed ultra-small triphenylphosphine-modified Ceria NPs, following coated with mPEG-TK-PLGA polymer and loaded atorvastatin, was successfully constructed and evaluated. Atv/PTP-TCeria NPs could passively target the kidney and release drug responding to high-level ROS and TCeria NPs target mitochondria to scavenge excessive ROS more effectively. Atv/PTP-TCeria NPs overcome the shortcomings of simple Ceria NPs, not only improve its stability and biocompatibility, endow the ability to target mitochondria, but also reduce the dose of ceria by loading atorvastatin and synergistically exerted anti-inflammatory and antioxidant effects. Therefore, this ROS-responsive nano-drug delivery system combining mitochondria-targeting ceria nanoparticles with atorvastatin will be a promising treatment for sepsis-induced acute kidney injury.

## Supplementary Material

Supplementary figures and tables.

## Figures and Tables

**Scheme 1:**
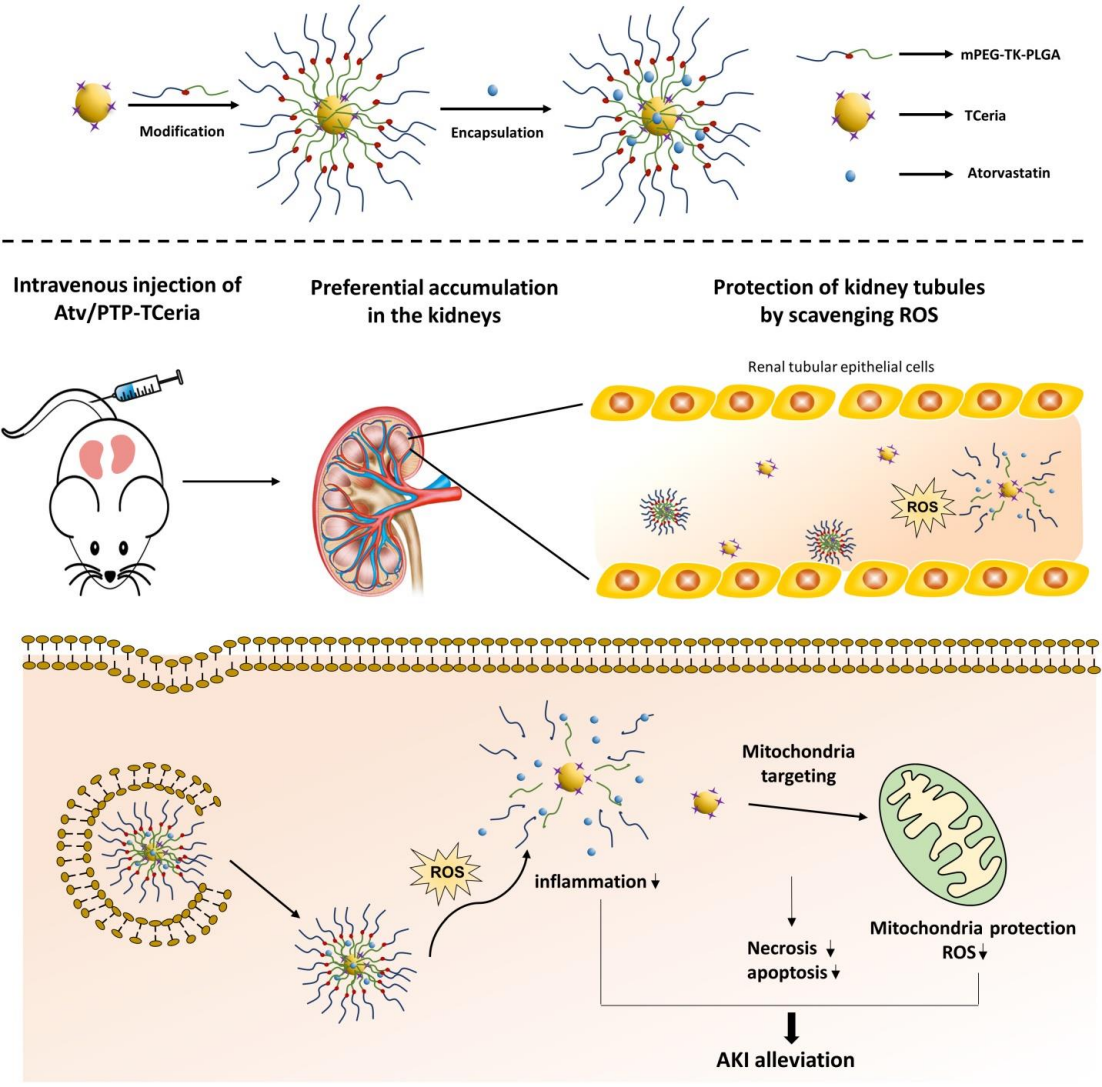
Schematic illustration of Atv/PTP-TCeria NPs for acute kidney injury. Ceria NPs are modified with triphenylphosphine (TCeria NPs) to be endowed the ability to target mitochondria, then TCeria NPs are coated by mPEG-TK-PLGA polymer and load atorvastatin. Atv/PTP-TCeria NPs could passively target the kidney and release drug responding to high-level ROS and TCeria NPs would target mitochondria to scavenge excessive ROS for ameliorating AKI.

**Figure 8:**
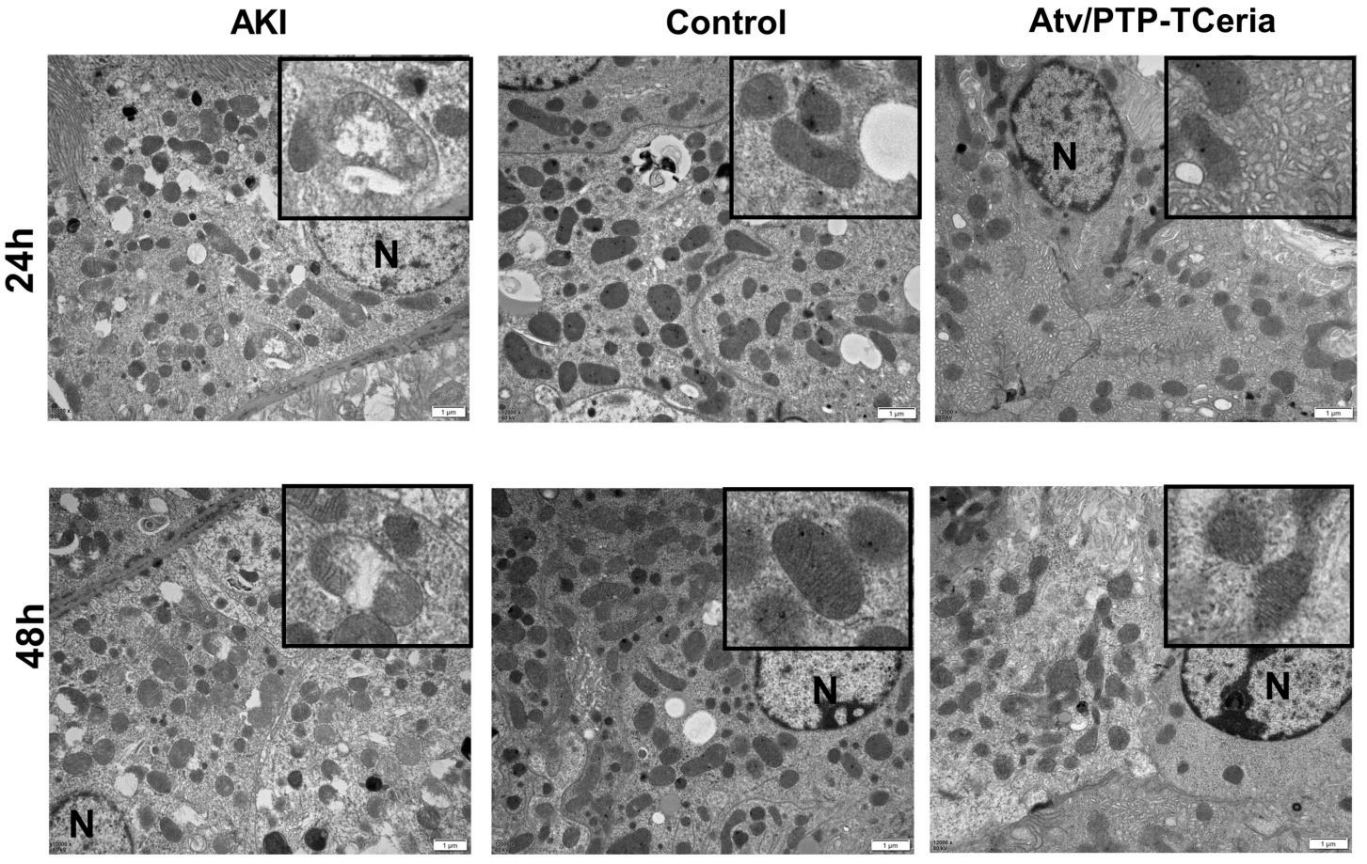
*In vivo* protective effect on mitochondrial structure of Atv/PTP-TCeria NPs. TEM images of mitochondria from representative samples in the control, AKI, and Atv/PTP-TCeria NPs treated groups (at 24 h and 48 h). Scale bar = 1 μm. N means nucleus.
