# Supplementary material for: ROS-responsive nano-drug delivery system combining mitochondria-targeting ceria nanoparticles with atorvastatin for acute kidney injury
Source: Theranostics. 2020 Jan 16;10(5):2342–57. doi: 10.7150/thno.40395 (PMC7019163; doi:10.7150/thno.40395)
Supplement: Supplementary file 1 — Supplementary figures and tables. [file thnov10p2342s1.pdf]

Supporting Information

ROS-responsive nano-drug delivery system combining mitochondrial targeted ceria nanoparticles with atorvastatin for acute kidney injury

*Hui Yu [1], Feiyang Jin [1], Di Liu [1], Gaofeng Shu [1], Xiao-Juan Wang [2], Jing Qi [1], Mingchen Sun [1], Ping Yang [2], Saiping Jiang [2,**], Xiaoying Ying [1], Yongzhong Du [1,*]*

Table S1: A glossary of abbreviations in this paper

**Full name**	**Abbreviation**
reactive oxygen species	ROS
methoxypolyethylene glycols	mPEG
poly(lactic-co-glycolic acid)	PLGA
thioketal	TK
superoxide dismutase	SOD
catalase	CAT
Malondialdehyde	MDA
Inductively coupled plasma mass spectrometry	ICP-MS
Nuclear magnetic resonance	NMR
2,7-Dichlorodi-hydrofluorescein diacetate	DCFH-DA
4',6-diamidino-2-phenylindole	DAPI
fluorescein isothiocyanate	FITC
propidium iodide	PI

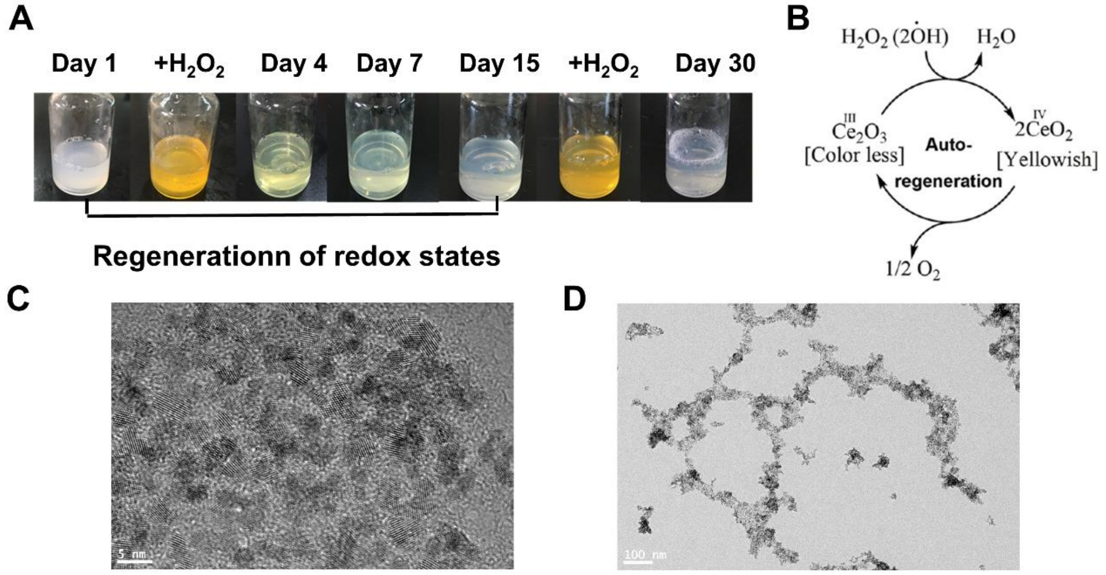

Figure S1. (A) Color changes of TCeria NPs on the addition of H_2O_2. These behaviors reflect the auto-regenerative nature of Ceria NPs. we added 0.1 M H_2O_2 to TCeria NPs. The color of the solution quickly changed to dark yellow (day 1). 15 days later, the color of the samples reverted back to colorless. we repeated the oxidation process with 0.1 M H_2O_2 additions on day 15 with the same samples. The color of the solution quickly changed to dark yellow again. (B) The plausible mechanism of free radical scavenging activity and auto-regenerative properties of Ceria NPs.[1] (C) Representative high-resolution TEM (HR-TEM) image of TCeria NPs showing uniform shape and size of TCeria NPs (~5 nm). Scale bar = 5 nm. (D) Representative TEM image of TCeria NPs which dispersed in water after natural drying and underwent ultrasonic treatment for 15 minutes, showing obvious agglomeration and good dispersion could not be obtained. Scale bar = 100 nm.

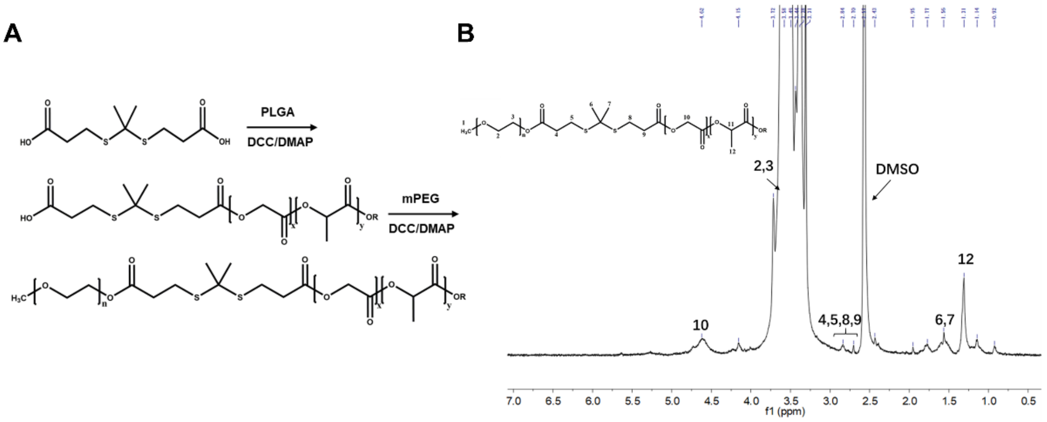

Figure S2. (A) The synthetic route of mPEG-TK-PLGA polymer. mPEG-TK-PLGA was synthesized by esterification of the carboxyl group of TK with the hydroxy group of mPEG and PLGA. (B) 1 H-NMR spectra of mPEG-TK-PLGA.

Table S2: Physicochemical characterization of atorvastatin-loaded PTP-TCeria NPs

Formulation	Drug/polymer	Size/nm	PDI	DL%	EE%	ζ-potential/mV
Atv-load	5%	37.5±2.62	0.373±0.017	3.01±0.02	60.4±0.381	-1.72±0.433
	10%	43.1±7.50	0.227±0.029	6.13±0.01	61.3±0.143	-4.45±0.751
	15%	50.7±6.68	0.346±0.034	9.68±0.03	64.6±0.789	-8.46±0.743
	20%	54.8±4.84	0.280±0.032	14.26±0.03	71.3±0.712	-19.1±1.18

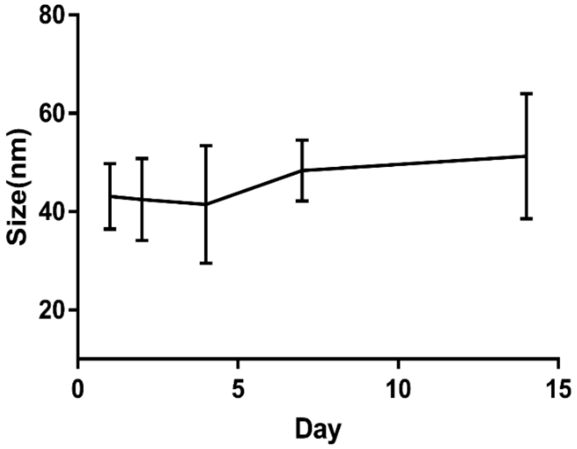

Figure S3. Hydrodynamic diameters of Atv/PTP-TCeria NPs in water for 14 days. the diameters of Atv/PTP-TCeria NPs were not significantly different after storing for 14 days at 4°C, showing Atv/PTP-TCeria NPs had good stability.

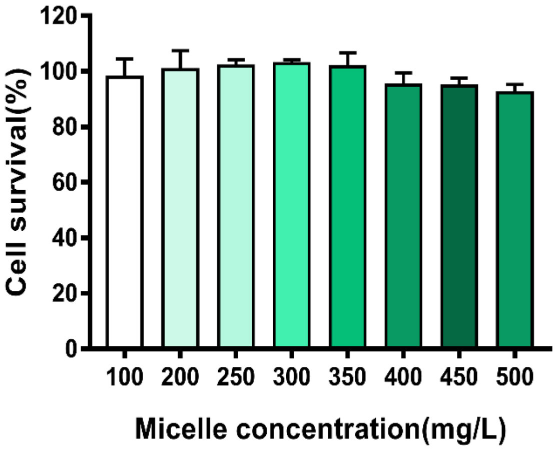

Figure S4. Cell viability of mPEG-TK-PLGA polymer in HUVECs after 24 h of exposure. mPEG-TK-PLGA polymer was nontoxic below 500mg/L.

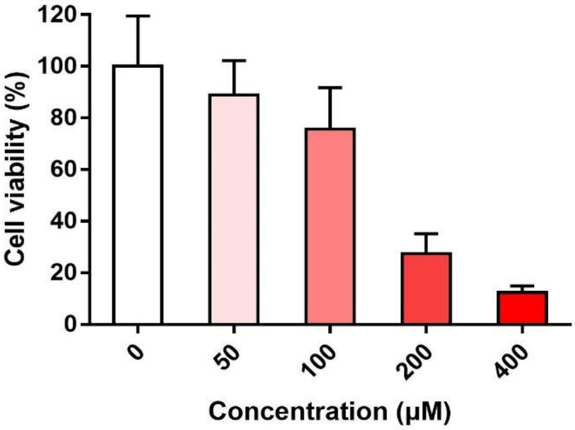

Figure S5. Cell viability in HUVECs after incubation with different concentrations of H_2O_2 for 24h.

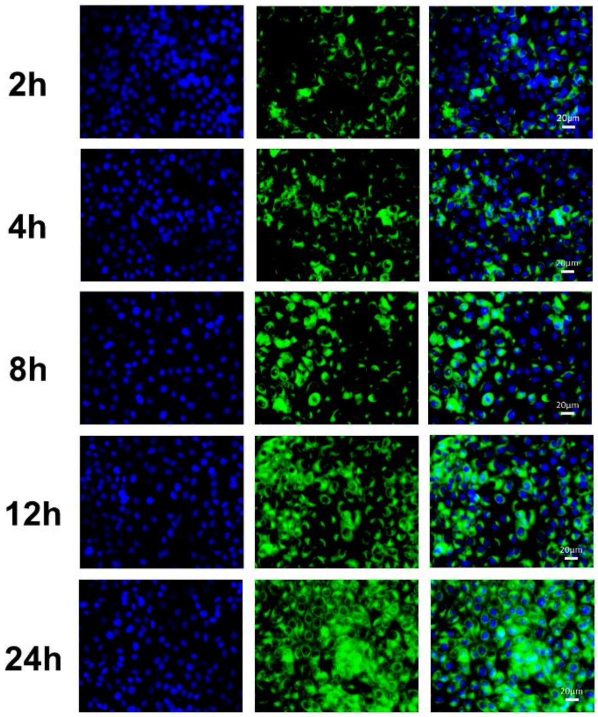

Figure S6. Fluorescence confocal microscope images of cellular uptake of Atv/PTP-TCeria NPs (scale bar = 20 μm).

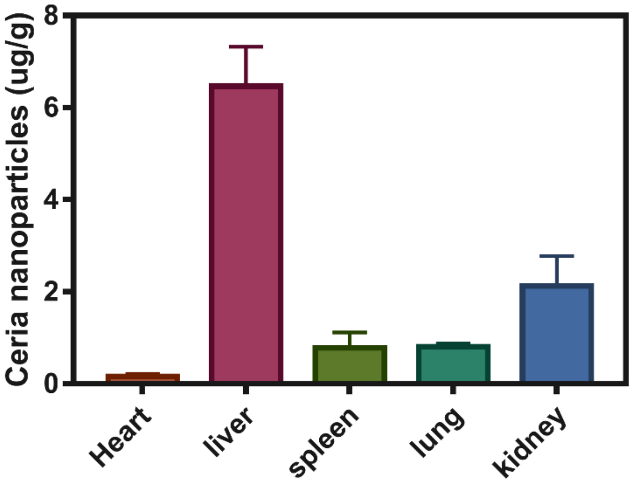

Figure S7. Bio-distribution of Ce per gram of tissues in the AKI mice main tissues (heart, liver, spleen, lung and kidney) of injection of 1 mg/kg Atv/PTP-TCeria NPs by intravenous administration after 6h. Method: AKI or healthy mice were injected intravenously with Atv/PTP-TCeria NPs at a dose of 1 $mg \cdot kg^{-1}$. The mice were sacrificed at 6h and major organs were collected. After drained the water and weighed the organs, we weighed exactly 20 mg of tissue and add nitric acid to digest thoroughly. The samples were filtered and measured by ICP.

Reference

1.Mitra RN, Gao R, Zheng M, Wu MJ, Voinov MA, Smirnov AI, et al. Glycol Chitosan Engineered Autoregenerative Antioxidant Significantly Attenuates Pathological Damages in Models of Age-Related Macular Degeneration. Acs Nano. 2017; 11: 4669-85.
